# Hierarchical event segmentation of episodic memory in virtual reality

**DOI:** 10.1038/s41539-025-00321-6

**Published:** 2025-05-11

**Authors:** Yue Li, Mikael Johansson, Andrey R. Nikolaev

**Affiliations:** 1 Faculty of Behavioural, Management and Social Sciences, University of Twente, Enschede, The Netherlands; 2 Department of Psychology, Lund University, Lund, Sweden

**Keywords:** Psychology, Human behaviour

## Abstract

Contextual shifts are crucial for episodic memory, setting event boundaries during event segmentation. While lab research provides insights, it often lacks the complexity of real-world experiences. We addressed this gap by examining perceptual and conceptual boundaries using virtual reality (VR). Participants acted as salespeople, interacting with customers in a VR environment. Spatial boundaries separated visually distinct booths, while conceptual boundaries were defined by customer requests. Memory was assessed through a recency discrimination task. Results indicated boundary crossings impaired sequence memory, consistent with previous findings. Crucially, conceptual boundaries, but not spatial boundaries, significantly influenced the accuracy of sequence memory, suggesting that top-down processes dominate bottom-up perceptual processes in naturalistic event segmentation. Confidence in correct responses indicated that perceived memory quality was highest when participants stayed within and did not cross both spatially and conceptually defined events. Findings highlight VR’s effectiveness for studying hierarchical contextual influences in interactive episodic memory tasks.

## Introduction

Episodic memory is a core component of human cognition, enabling the vivid recollection and reliving of personal experiences within their specific contexts^1^. Contextual information provides the framework within which events are encoded, stored, and retrieved^1–4^. During encoding, the content of the experience (what happened) is integrated with its context (where, when, and under what circumstances it happened), forming a coherent representation of the event. When retrieving episodic memories, the brain may use content-based cues to recall specific details of the event and context-based cues to reconstruct the broader context in which it occurred. The segmentation of events relies heavily on the contextual information surrounding them. According to event segmentation theory, episodic memory is structured by event boundaries that emerge from contextual shifts^5,6^. The perceptual system continuously generates predictions about ongoing events based on past experiences, expectations, and current context. Contextual shifts disrupt these predictions, causing prediction errors and establishing contextual boundaries. These boundaries mark transitions between event models, segmenting continuous experience into sequences of meaningful and structured experiences^7–9^.

Boundaries provide natural cues for the sequential organization of events, shaping the temporal structure of episodic memory. Memory representations develop gradually, leading to memories of experiences in close succession to share more similar characteristics, such as background contextual information, compared to those further apart^10–12^. Event boundaries serve as reference points for remembering the order in which events occurred, thereby establishing temporal order memory^8,13,14^. During retrieval, event boundaries function as anchors for reconstructive memory processes, such as recency judgments, by guiding the recall of the temporal order of items. The accuracy of recency judgments relies on whether these items occur within or across event models. Items within an event model are more strongly associated because their temporal context is well-defined, enhancing accurate encoding and recall of the order of the items^13,15,16^. In contrast, the presence of an event boundary between two items during encoding negatively impacts order memory^17,18^. Thus, remembering the order of items becomes more challenging when separated by an event boundary, as the boundary disrupts the flow of events and hinders the formation of a coherent event sequence.

The effects of event boundaries on recency judgments are complex and depend on a number of factors, particularly the type of event boundary^5,15,19^. Event segmentation theory proposes that the shifts in different types of context lead to distinct event boundaries. Changes in motion, location, color, and sound result in *perceptual* boundaries. For example, shifts in location create spatial boundaries^7,9,20^, similar to physical markers of location separation, such as doors, walls, etc.^21^. Changes in expectations, goals, and task sets form *conceptual* boundaries, often linked to shifts in an individual’s cognitive or attentional focus^7,22^. The combination of perceptual and conceptual boundaries shapes the hierarchical structure of events in time^6,7^. Perceptual boundaries delineate lower-level or sub-events at small temporal scales by capturing environmental changes. Conceptual boundaries represent overarching themes and global shifts in goals or context that delineate higher-level events at large temporal scales. The relative importance of perceptual and conceptual boundaries for event segmentation may vary based on the nature of ongoing activities and individual goals: in some situations, perceptual boundaries may take precedence in organizing events, while in others, conceptual boundaries may take precedence. Consequently, the primacy of one type of boundary over another may shift dynamically as new information emerges during event perception. This flexibility allows for adaptive event segmentation that supports goal-relevant thinking and behavior.

In everyday life, contextual information is rich and diverse, encompassing a wide range of multimodal sensory inputs, situational variables, and social interactions. However, psychological research conducted in controlled environments often fails to capture the full complexity of mental processes as they occur in the real world^23,24^. This gap highlights the need for experimental conditions that more accurately reflect real-life situations. In particular, episodic memory research faces challenges in replicating the intricate interplay of spatial, temporal, situational, and social factors that characterize real-world memory experiences. Traditional laboratory experiments often simplify and control these variables to isolate specific effects, eliminating the unpredictability and variability inherent in natural environments. This approach also overlooks the integration of multimodal cues from multiple sensory inputs and neglects the cumulative impact of repeated exposure to complex contexts over time. These limitations may lead to an oversimplified understanding of how episodic memories are encoded, stored, and retrieved in real-world scenarios and may fail to fully reflect the naturalistic process by which events are integrated and segmented into memory.

Previous studies have addressed these concerns by using videos of real people performing everyday tasks. For example, participants viewed a video of a car door assembly task and were instructed to segment this activity into fine and coarse event units. The identified event boundaries revealed how participants perceived the hierarchical structure of the task. Repeated training improved memory specifically for broader, coarser-level steps rather than finer details, highlighting the hierarchical nature of event segmentation during practical skill acquisition^25^. Similarly, another study used videos of sitcom episodes, demonstrating that shifts in situation models during viewing influenced memory and predictive accuracy at event boundaries. The findings suggested that updates to event models occurred incrementally rather than globally^26^. While these highly realistic experiments confirm that event segmentation mechanisms apply beyond artificial laboratory settings and remain relevant in real-world contexts, they still lack the interactive component necessary for authentic episodic memory formation. Interactivity enables the establishment of causal links between actions and outcomes, and perceiving these links facilitates the integration of discrete experiences into a cohesive sequence of events, which is an essential component in the formation of episodic memories^5,27,28^. This interactivity is a central advantage of virtual reality (VR), making it a particularly powerful tool for investigating episodic memory. VR allows for the replication of real-life conditions from a first-person perspective, offering rich multisensory stimulation and opportunities for social interaction^16,29–31^. Unlike traditional laboratory experiments, which often lack the naturalistic context of learning and remembering, VR can more accurately mimic environments where event segmentation occurs naturally by incorporating complex, dynamic contexts and flexible task demands. Despite its potential, to our knowledge, only one VR study has explored the effects of event boundaries on episodic memory segmentation^16^. In that study, participants navigated adjacent rooms distinguished by wallpaper color and flooring while categorizing objects as “natural” or “man-made.” Temporal memory for object sequences was later assessed, comparing memory for objects encountered in the same room with those separated by a boundary. The findings revealed that spatial boundaries significantly influenced temporal memory, with better recall for objects encountered within the same room than those separated by a boundary.

In real-life event segmentation, spatial and conceptual boundaries naturally co-occur, yet their combined influence on memory formation remains underexplored. Previous research examining the interactive effects of these boundaries has primarily relied on imagined scenarios, such as associating celebrity faces with spatial (e.g., rooms) and non-spatial (e.g., desserts) contexts^32^. This study found that conceptual similarity facilitated temporal memory equally across spatial and non-spatial contexts, suggesting no significant difference in how these boundary types influence memory organization. However, because the paradigm relied on imagination rather than embodied interaction, it may not fully capture how concurrent spatial and conceptual boundaries shape event segmentation in real-world experience. Thus, a gap remains in understanding how these boundaries interact during memory formation in naturalistic environments. To address this, our study employs a VR-based approach adapted from Horner and colleagues^16^ to investigate how spatial and conceptual boundaries influence event segmentation and memory in immersive scenarios designed to closely mimic real-life contexts.

**Fig. 1:**
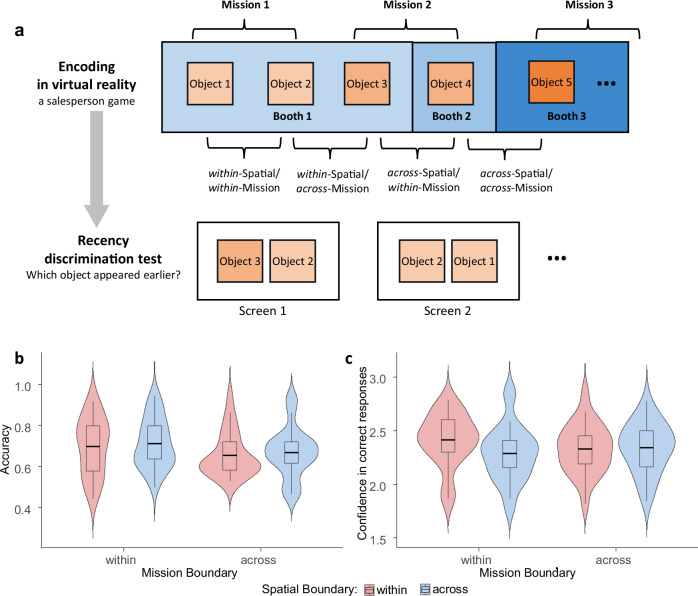
The design and results of the experiment. **a** The general scheme of the experiment and four combinations of boundary conditions. **b** Memory accuracy in the recency discrimination test for the four spatial and mission boundary levels. **c** Confidence in correct responses in the recency discrimination test for the four spatial and mission boundary levels. The violin plots illustrate the distribution, density, and spread. The box shows the interquartile range (IQR) with the mean as a thick horizontal line; whiskers represent values within 1.5 times the IQR.

We designed a VR memory encoding task, followed by a subsequent memory assessment outside VR (Fig. 1a). During encoding, participants acted as salespersons interacting with customers across different booths. Their primary task was to remember the order in which objects appeared and to deliver them to waiting customers. The conceptual (mission) boundaries were defined by interactions with different customers, and the spatial boundaries were defined by booth environments. These variations created four boundary conditions: 2 × 2 combinations of within/across mission and within/across spatial boundaries. After completing the VR encoding session, participants performed a recency discrimination test on a computer. This test required them to identify which of two objects appeared earlier during the VR session and to indicate their confidence in each decision. Based on event segmentation theory^6,7,33^, we predict distinct effects of mission and spatial boundaries on temporal order memory. Specifically, mission boundaries characterized by shifts in task goals, cognitive focus, or attentional demands are expected to strongly disrupt the coherence of event models^26^. These conceptual shifts are hypothesized to weaken the formation of temporal associations between items encountered across boundaries, thereby significantly impairing the accuracy of recency judgments^6,7^. While spatial boundaries have also been shown to disrupt the continuity of event representations, their impact on temporal associations may be comparatively weaker. For instance, the presence of a spatial boundary at encoding (e.g., a doorway between rooms) has been shown to impair participants’ ability to remember the order of presented objects^16,18^. However, in our salesperson task, changes in cognitive goals are likely more salient than shifts in physical location. Therefore, we expect mission boundaries to exert stronger negative effects on temporal order accuracy than spatial boundaries, reflecting their more profound disruption of episodic encoding. By investigating the interplay between these boundary types, we aim to enhance our understanding of their role in shaping the hierarchical organization of episodic memory.

## Results

### Accuracy

First, we estimated the temporal order memory for the object order encoded during the VR session, which was assessed in the following recency discrimination test; the mean accuracy across participants was 68.4% (SD = 10.2%).

Next, we estimated the response accuracy as a function of the four combinations of boundary types using a generalized linear mixed-effects model (GLMM) with the formula:

model_accuracy ← glmer (accuracy ~ Mission boundary * Spatial boundary + blocks + (1 | subid) + (1 | subID:objectID) + (1|repetition), dataset, family = binomial(link = “logit”))

Figure 1b shows the distribution of mean accuracy in the recency discrimination test. The overall *R*^2^ of the accuracy model was 5.3%, indicating how the combined fixed and random effects explain the variability in the binary outcome (accuracy). The fixed effect *R*^2^ was 0.57%, which indicates the proportion of variability in accuracy explained by fixed effects alone. The variance of random effects was 4.7%, which accounts for the variability in accuracy among participants.

We found a significant main effect of crossing the mission boundary (*p* = 0.04), indicating better memory when participants were within the mission than when they crossed the mission boundary. There was no significant effect of crossing the spatial boundary or interaction between the boundary conditions. Furthermore, accuracy significantly increased with the block order (*p* < 0.001).

To assess the contribution of the random effects to the goodness of fit, we tested the likelihood ratio by comparing the fit of a model with and without random effects. As shown in Table 1, the full model showed a significantly better fit than the model without object repetition (*p* = 0.01) and *subID* (*p* < 0.001). Thus, the selected random factors significantly improved the model fit.

**Table 1:** Likelihood ratio test for the accuracy model with-/without-random effects

	N-par	AIC	BIC	Log Lik	Deviance	*χ*²	*df*	*p*-value
Full model	8	4454	4504	−2219	4438			
Model without repetition	7	4459	4502	−2222	4445	6.29	1	0.01*
Model without *subID*	7	4560	4603	−2273	4546	107.59	1	<0.001***
Model without *subID:objectID*	7	4452	4496	−2219	4438	0	1	0.9

*p*-Values are based on Wald-type tests for fixed effects. Statistical significance is indicated as follows: *p* < 0.001 (***), *p* < 0.01 (**), *p* < 0.05 (*).

To ensure that the effect reported above is not explained in terms of different inter-object intervals between experimental conditions, we conducted a planned comparison test in which we excluded the *within-*Mission/*within-*Spatial level with the shortest interval and the *within-*Mission/*within-*Spatial level with the longest interval from the model. The reduced model, including only levels with comparable inter-object intervals (*within-*Mission/*across-*Spatial level and *across-*Mission/*within-*Spatial level), showed significantly higher accuracy in the *within-*Mission/*across-*Spatial than in the *across-*Mission/*within-*Spatial level (estimate = 0.28, Std.Error = 0.1, *Z*-value = 2.7, *p* = 0.007).

### Confidence

To better understand participants’ metacognitive processes, we analyzed their response confidence. We selected only the test trials with correct responses. Participants gave 21% “unsure”, 34% “fairly sure”, and 45% “quite sure” responses. We excluded one outlier participant who responded “unsure” 90% of the time, even though their accuracy was high enough (~60%).

To evaluate the effect of the boundary conditions on confidence, we used a general linear mixed-effects model with the following formula:

model_confidence ← lmer (confidence ~ Mission boundary * Spatial boundary + blocks + (1 | subid) + (1 | subID:objectID) + (1| repetition), data subset with only correct responses)

Figure 1c shows the distribution of mean memory confidence for correct responses in the recency discrimination test. The overall *R*^2^ of the confidence model was 71%, indicating how the combined fixed and random effects explain the variability in the confidence reports.

We found a marginally significant main effect of crossing the mission boundary (*p* = 0.055) on confidence, indicating higher confidence in correct responses when participants were within the mission boundary compared to when they crossed the mission boundary (Table 2). We also found a significant main effect of crossing the spatial boundary (*p* = 0.004), indicating higher confidence when participants were within the spatial boundary compared to when they crossed the spatial boundary. Importantly, however, there was an interaction between the boundary conditions (*p* = 0.03). The post-hoc comparisons of marginal means with the Sidak correction revealed higher confidence for the *within-*Spatial and *within-*Mission boundary conditions than for the across-spatial and *within-*Mission boundary conditions (*t* = 2.9, *p* = 0.02). Furthermore, confidence significantly increased with the block order (*p* = 0.009).

**Table 2:** Fixed effects estimate of the confidence model

	Estimate	Std. Err	*t*	*p-*value	
(Intercept)	2.30	0.07	34	<.001	***
Mission boundary	−0.08	0.04	−1.9	.055	.
Spatial boundary	−0.19	0.04	−2.9	.004	**
Mission boundary * Spatial boundary	0.13	0.06	2.2	.03	*
blocks	0.02	0.01	2.6	.009	**

*p*-Values are based on Wald-type tests for fixed effects. Statistical significance is indicated as follows: *p* < 0.001 (***), *p* < 0.01 (**), *p* < 0.05 (*).

We compared the fit of a model with random effects to a model without random effects. As shown in Table 3, the full model showed a significantly better fit than the model without object repetition (*p* = 0.01) and subID (*p* < 0.001). Thus, the selected random factors significantly improved the model fit.

**Table 3:** Likelihood ratio test for the confidence model with-/without-random effects

	N-par	AIC	BIC	Log Lik	Deviance	*χ*²	*df*	*p*-value
Full model	9	5363	5415	−2672	5345			
Model without repetition	8	5364	5411	−2674	5348	3.55	1	0.059
Model without *subID*	8	5480	5526	−2731	5464	118.83	1	<0.001***
Model without *subID:objectID*	8	5361	5408	−2673	5346	0.63	1	0.4

*p*-Values are based on Wald-type tests for fixed effects. Statistical significance is indicated as follows: *p* < 0.001 (***), *p* < 0.01 (**), *p* < 0.05 (*).

## Discussion

We investigated the segmentation of episodic memory events under experimental conditions designed to mimic real-life, interactive scenarios using VR simulation. During the encoding session, participants assumed the role of a salesperson, navigating through adjacent booths and delivering various products (objects) to different customers. They were tasked with memorizing the order in which the objects appeared. Events were defined by two types of boundaries: spatial boundaries, which were delineated by the identities of the booths, and mission boundaries, determined by the identities of the customers. Following the encoding session, a recency discrimination test conducted outside of VR assessed participants’ temporal order memory for objects’ appearances during encoding. Our findings indicate that spatial boundaries did not generally affect memory performance, whereas mission boundaries significantly impacted the *accuracy* of temporal order memory. Moreover, we observed that staying within the same spatial event context enhanced participants’ *confidence* in the correct memory reports. These results suggest that, within the context of our task, mission boundaries contribute more prominently to the segmentation of events crucial for episodic remembering.

In our study, the mission boundaries signify a conceptual shift related to the task goal, and the spatial boundaries signify a perceptual shift related to the physical environment. Therefore, the observed effect of mission boundaries on the *accuracy* of temporal order memory indicates that in the task where perceptual and conceptual boundaries coexist, conceptual shifts play the primary role in event segmentation. This finding is consistent with the event segmentation theory, which posits the dominance of top-down (cognitively driven) over bottom-up (sensory-driven) processes in the hierarchical structuring of events^6,7^.

The analysis of *confidence* in correct responses enhances understanding of the accuracy results. While accuracy represents the objective correctness of a memory, confidence reflects participants’ subjective evaluation of that memory. In our study, confidence in correct responses reflects the perceived quality of accurate memories. This allowed us to assess subjective evaluations of the strength and robustness of memories, consistent with the prevailing understanding of confidence measures in memory research^34,35^. We observed that participants exhibited higher confidence when they stayed within both spatial and mission events than when they crossed these boundaries. We also found an interaction between mission and spatial boundaries, because confidence was higher within than across spatial boundaries in the within-mission condition. These findings indicate that the perceived memory quality is higher when encoding occurs within both spatial and mission events compared to encoding across these boundaries.

The differential effects of conceptual and spatial boundaries on accuracy and confidence suggest that these measures reflect distinct mechanisms underlying episodic memory formation. When a conceptual boundary is crossed, the thematic or narrative continuity of the event is interrupted, making it more difficult for participants to bind elements together into a coherent episodic representation. In contrast, crossing a spatial boundary disrupts the perceptual continuity of the event, which may reduce the subjective sense of fluency or vividness during retrieval. Thus, perceptual continuity plays a role in subjective memory strength, as reflected in confidence, even when it does not significantly affect objective accuracy.

For both accuracy and confidence, we observed the detrimental effect of boundary crossing, which is consistent with previous research indicating better sequence memory in serial encoding for within-event contexts compared to cross-event contexts^7,16,36^. Crossing an event boundary impairs sequence memory for stimuli on either side of the boundary and increases the subjective temporal distance between stimuli^13,17^. This may happen because staying within both boundaries supports the formation of a coherent event with strong associations between its elements, while crossing boundaries disrupts the unfolding of the event and impairs between-element associations^37,38^.

Although our findings align with established knowledge regarding the role of various boundary types in event segmentation, they are notable for being obtained in a VR paradigm providing a more naturalistic and interactive experimental setting than traditional laboratory tasks. While VR has rarely been employed in the study of event segmentation and memory, our findings align with a prior VR study examining the effects of spatial boundaries^16^, as well as with similar results from laboratory-based research presenting stimuli on a computer screen^5,15,19^. Notably, we observed an interactive effect of spatial and conceptual boundary crossings on temporal order memory—an effect not identified in previous laboratory studies that relied on participants’ imagination, e.g.,^32^ This discrepancy suggests that the interactivity and ecological realism afforded by VR significantly shape event segmentation, highlighting the unique advantages of immersive methods for investigating episodic memory processes under conditions that closely resemble real-world experiences.

Episodic memory in real-world contexts is shaped by numerous interacting factors, including the unpredictability and variability of contextual information, its inherently multimodal nature, and the uncertain outcomes resulting from interactions between agent actions and environmental components. This complexity plays a critical role in how memories are formed. However, fully capturing these dynamics remains challenging in current VR environments, which often rely on simplified settings and artificial characters, such as those used in our study. Despite these limitations, VR environments offer substantial advantages in terms of rigorous experimental control and the ability to systematically and with precision manipulate boundary conditions that are difficult to achieve in natural settings. In addition, the primary advantage of VR in psychological research lies not in its realism but in its capacity for interactivity, which demands active engagement from participants with both tasks and environments. Our VR paradigm was therefore designed to emphasize high levels of interaction with both the virtual environment and characters, positioning interactivity as a central feature in investigating episodic memory formation. Nevertheless, future studies incorporating more realistic scenes and socially dynamic characters will be essential to assess the generalizability of these findings to everyday memory processes.

The presence of multiple adjacent booths in our VR setup introduced a degree of spatial complexity that may have influenced memory formation. Spatial boundaries can be operationalized in various ways, each offering distinct advantages and limitations. Future research could explore how various manipulations of spatial complexity (e.g., fewer locations or simpler spatial transitions) can influence memory encoding and retrieval. Such experiments would help clarify the specific role of spatial context in shaping episodic memory formation. Furthermore, although conceptual and perceptual boundaries were balanced in number and complexity, the salesperson’s task inherently emphasized customer-related goals, potentially making conceptual boundaries more salient. Although goal relevance naturally guides human behavior, future studies could explore strategies to better balance conceptual and perceptual elements by introducing strategies that highlight perceptual boundaries. For instance, tasks could incorporate stronger interaction with spatial components, such as requiring active navigation or active object search and retrieval across multiple locations, thereby enhancing the representation of perceptual boundaries.

As outlined in Methods, the temporal intervals between handling two consecutive objects during encoding depend on the boundary conditions. The interval was shortest when two objects appeared one after the other within the *within-*Spatial*/within-*Mission level and longest when separated by moving to another booth and meeting a new customer in the *across-*Spatial/*across-*Mission level. While these variations in inter-object intervals may influence temporal order memory, our results did not provide evidence for such an effect. The mean accuracy remained similar across the *across-*Spatial/*within-*Mission level with the mid-long interval and in the *within-*Spatial/*within-*Mission level with the shortest interval (Fig. 1b). Moreover, if differences in memory accuracy across conditions were primarily driven by variations in inter-object intervals, then we would expect accuracy to differ significantly when comparing the conditions with the shortest interval (*within*-Spatial/*within*-Mission) and longest interval (*across*-Spatial/*across*-Mission). Such differences would be statistically manifested as an interaction between spatial and mission boundaries, since these two factors define the extremes of inter-object intervals in our design. However, we found no interaction (*p* = 0.97). Furthermore, after excluding from the model the levels with the shortest (*within-*Spatial/*within-*Mission) and the longest (*across-*Spatial/*across-*Mission) inter-object intervals, memory accuracy was significantly higher in the *within-*Mission/*across-*Spatial than in the *across-*Mission/*within-*Spatial level (*p* = 0.007). Thus, even when inter-object intervals were made comparable, crossing mission boundaries impairs memory accuracy more than crossing spatial boundaries, supporting our main conclusions. These findings indicate that forming memories for temporal order under interactive conditions, involving a variety of boundary types, is a complex process not solely determined by the temporal gaps between encountered objects. However, future VR research on event segmentation could systematically control these temporal intervals, allowing researchers to more precisely isolate and examine the effects of different boundary types on memory performance.

Our study underscores the potential of VR as an effective tool in memory research, offering new insights into how event segmentation shapes episodic memory. It opens promising avenues for further exploration of how personal experiences are structured and remembered. Future VR research could build on these findings by varying task complexity, integrating realistic physical movement, and exploring additional sensory modalities. Moreover, capturing more comprehensive behavioral data during VR experiments, such as exposure durations, interaction positions, and eye-tracking metrics, could provide deeper insights into the cognitive mechanisms underlying event segmentation.

More generally, the application of VR in cognitive psychology overcomes the limitations of traditional laboratory-based experimental designs, including the lack of agency and embodiment, which hinder the faithful representation of cognition in the real world. Additionally, the VR approach reduces the challenges of artificial and decontextualized environments and passive participant behavior that are common in laboratory paradigms^23,24^. Thus, the application of VR expands the methodological repertoire and offers new opportunities to push the frontiers of understanding cognition and behavior in complex and interactive real-world settings.

## Methods

### Participants

26 healthy adult volunteers (9 males, *M*_age_ ± SD = 25.5 ± 2.4) participated in the study. All participants had normal or corrected-to-normal vision, no reported history of neurological or psychiatric disease, and no history of cybersickness. The study was conducted in accordance with the Declaration of Helsinki, the Swedish Act concerning the Ethical Review of Research involving Humans (2003:460), and the local ethical guidelines at Lund University. All participants gave informed written consent. In the experimental session, single blocks were excluded after data collection for three participants: one block was excluded because a participant failed to concentrate on the task, and two blocks were excluded due to hardware malfunction.

### Experiment design and procedure

**Fig. 2:**
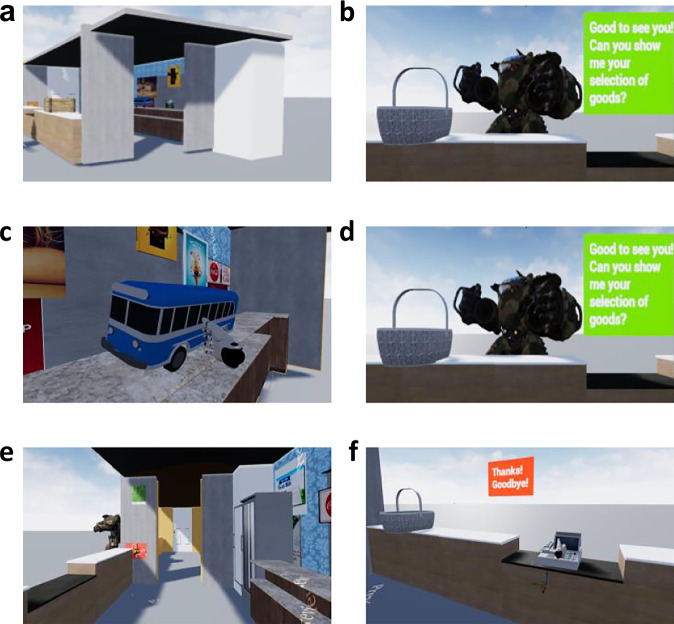
The main steps of the VR salesperson game (screenshots from a first-person perspective during gameplay). **a** The participant started near the entrance of the booth. **b** Participants looked at the customer for 3 s to start the mission. **c** Participants picked up the requested object from the shelf in the back. **d** Participants put the object into the basket on the counter next to the customer. **e** The customer moved to the adjacent booth on the right, the door opened, and the participant walked through the door into the next booth. **f** The cash register appeared when the customer had received all the objects. To end the current mission, the participant had to touch the cash register, triggering a “thank you” message, after which the customer left (and the new customer appeared).

The experiment consisted of six blocks, each including a memory encoding VR session and a desktop recency discrimination test outside VR. In the VR session, participants played the role of a salesperson who had to serve multiple customers in several adjacent booths. At the beginning of the VR session, participants were positioned near the entrance of the first booth. They then entered the booth and made their way to the counter (Fig. 2a). In front of the counter, a customer was waiting for service. Participants looked at the customer for 3 s until the green dialog box appeared, indicating the start of a mission (Fig. 2b). During the mission, various objects appeared one by one on the shelves behind the participant (Fig. 2c). A random number of objects, ranging from one to three, appeared in each mission. Participants were instructed to remember the sequential order in which the objects appeared for the subsequent recency discrimination test. They were instructed to pick up the objects from the shelves and place them in the basket on the counter (Fig. 2d). The next object requested by the customer appeared in either the current booth or the adjacent booth. The adjacent booth was located to the right of the front counter and connected by a door. The customer either waited in the current booth or moved to the front of the adjacent booth (Fig. 2e). When the customer moved to the adjacent booth, the door connecting the two booths opened automatically. Each booth had a unique appearance, distinguished by different wall colors and varying banners and posters displayed on the walls. The different decorations of each booth are thought to be implicitly remembered, thus increasing the temporal separability of sequentially encountered objects in memory across spatial boundaries. A sound was emitted whenever participants crossed the doorway, marking the boundary between booths. Upon successfully delivering all the requested objects, a beep indicated the completion of the handover, and the cash register appeared on the front counter. Participants were required to touch the register to finalize the mission. The sound of the cash register was accompanied by a red dialog box displaying a “thank you” message, indicating the customer’s satisfaction (Fig. 2f). Subsequently, a new customer appeared either in front of the counter in the current booth or in the adjacent booth (the video showing the main parts of the experiment is available at https://osf.io/u7rzw).

The VR environment was custom-built using Unreal Engine 4.26, compatible with Steam VR. It was presented using a full HTC Vive Pro 2 kit, powered by an NVIDIA GeForce GTX 1080 graphics card.

**Fig. 3:**
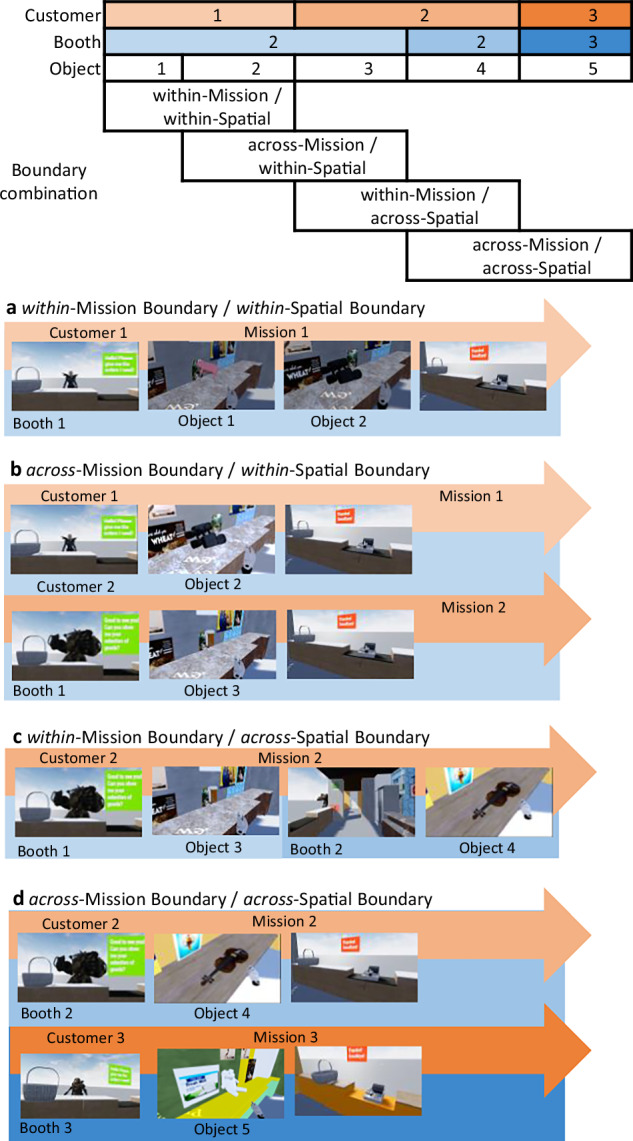
A scheme of four levels of the experimental conditions. **a** *within* mission / *within* spatial: two objects are handed to the same customer in the same booth. **b** *across* mission / *within* spatial: two objects are handed to different customers in the same booth. **c** *within* mission / *across* spatial: two objects are handed to the same customer in different booths. **d** *across* mission / *across* spatial: two objects are handed to different customers in different booths.

The contexts of object encoding provided four levels of boundary conditions for participants within the VR environment. These levels were defined by whether the participants remained within or crossed mission or spatial boundaries while handling objects. The levels were combined in a 2 × 2 experimental design with the factors Mission boundary (within vs. across) and Spatial boundary (within vs. across) (Fig. 3). The identity of the customer defined the mission boundaries; that is, if a subsequent object was intended for the same customer, the participant was maintaining the mission, and this level was referred to as *within-mission*. If the subsequent object was requested by a different customer, the participant started a new mission, and this level was referred to as *across-mission*. The identity of the booth, distinguished by different contextual features, defined the spatial boundaries. If a subsequent object was available in the same booth, the participant remained in the same space, and this level was referred to as *within-spatial*. If a subsequent object instead was available in a different booth, the participant was required to change space, and this level was referred to as *across-spatial*. Thus, the four experimental levels of our boundary manipulation were constructed as follows: (1) *within*-Mission/*within*-Spatial, (2) *across*-Mission/*within*-Spatial, (3) *within*-Mission/*across*-Spatial, and (4) *across*-Mission/*across*-Spatial.

Each block consisted of 13 different customers, 13 distinct booths, and 25 unique objects, totaling 150 objects across six blocks. Each of the four boundary levels was presented six times within each block, resulting in 144 appearances of boundary levels across the six blocks. Each block of the main experiment lasted approximately 15 min. To mitigate motion sickness, participants were seated in the VR session and allowed to rest between blocks for as long as they needed, with typical breaks of 5–10 min. The main experiment was preceded by a practice session with 16 objects in the VR environment, with all boundary conditions and 15 memory tests. Participants received feedback on the correctness of their responses after each test and were informed of the total correctness score at the end of the practice. The total duration of the practice and main experiment was approximately 135 min, including 20 min for practice, 90 min for six blocks, and 25 min for breaks.

### Recency discrimination test

**Fig. 4:**
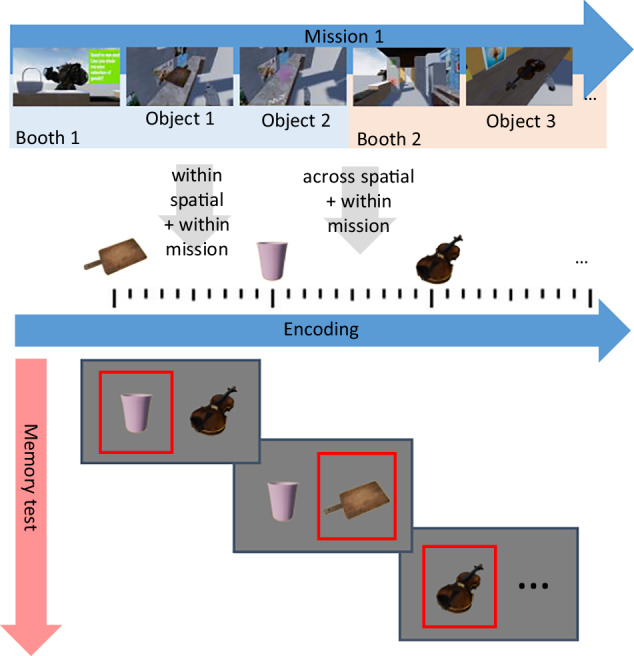
A scheme of encoding a sequence of objects in a VR scene and presenting the corresponding objects in the recency discrimination test. The recency discrimination test outside of VR for objects 1, 2, and 3 assessed the effect of crossing these boundaries on temporal memory. Objects were presented in pairs on the computer screen, and the participant indicated which object (left or right) appeared earlier during VR encoding. The correct answer in each test is marked in red. In this example, the participant handed over objects 1 and 2 one after the other to a customer in Booth 1. Then, the participant went to Booth 2 and handed over object 3 to the same customer. Since objects 1 and 2 appeared in the same booth and were delivered to the same customer, the boundary condition level for these two objects was “within mission boundary, within spatial boundary”. Objects 2 and 3 appeared in different booths and were delivered to the same customer, thus, the boundary condition level for these two objects was “within mission boundary, across spatial boundary”.

To examine boundary effects on memory for object order in the encoding VR session, we used the recency discrimination test^39^. The test assessed memory for the object’s appearance in the encoding sequence. In each block, after the VR session, participants took off the VR headset and moved to the laptop computer, where they completed the test. On each test screen, two isolated objects, appearing in succession in the VR session, were presented side by side on a gray background (Fig. 4). Participants were asked to indicate which of the two objects appeared earlier in the VR object sequence by pressing the left or right arrow key. The left/right position of the earlier (target) object on the screen was randomized across trials. On the next screen, participants were asked about the confidence of their response (“unsure”, “fairly sure”, “quite sure”), which they had to rate by pressing the “left arrow”, “down arrow”, and “right arrow” keys, respectively. The test was self-paced with a 0.5-s fixation interval before each test trial and a 10-s response timeout. Each object was presented twice, once as a target and once as a distractor, except for the first object in the VR session, which served only once as a target. Thus, one block included 24 memory test trials. In six blocks, there were a total of 144 memory tests. The test was programmed with PsychoPy 2022.2.4^40^.

At encoding, participants interacted with objects by locating, grasping, holding, and placing them in a basket. The time interval between interacting with two successive objects varied according to a participant’s movement speed and boundary conditions. Specifically, the interval was shortest in the within-Mission/within-Spatial level, where two successive objects were handed to the same customer in the same booth. Each *across-*Mission level added about 6 s to that level, including 3 s to touch the cash register and 3 s to engage with a new customer to initiate the subsequent mission. Each *across-*Spatial level also added about 6 s to the within-Mission/within-Spatial level, including 3 s to open the door and 3 s to move to the adjacent booth. The longest interval occurred in the across-Mission/across-Spatial level, which combined both “across” levels. These variations in the intervals between objects may bias the accuracy of the temporal order memory, as objects encountered closer in time are generally remembered more accurately than those encountered at longer intervals^2,41^. Horner et al.^16^ encountered a similar challenge in their main experiment, where the intervals and path distances between objects were shorter within than across spatial boundaries. To estimate the influence of this factor on memory, they conducted another experiment where they increased the size of the rooms, thus lengthening the distances within boundaries than across them. However, these manipulations did not alter the original finding, demonstrating the robust nature of the boundary effect: crossing a spatial boundary decreased memory accuracy for the sequence of objects relative to staying within boundaries. In our current study, we integrated spatial and mission boundaries to reflect natural behavior in real-life situations. However, this integration made it a challenge to equalize the inter-object intervals in the experimental design. Consequently, we adopted a data-driven approach to monitor potential correlations between temporal order memory and inter-object intervals across the four boundary conditions. In the control analysis, we compared order memory for *within*-Mission/*across*-Spatial and *across*-Mission/*within*-Spatial levels, where inter-object intervals were of similar durations, excluding the *within*-Mission/*within*-Spatial level with the shortest interval and the *across*-Mission/ *across*-Spatial level with the longest interval from the model.

### Statistical analysis

To examine how accuracy and response confidence in the memory test depended on whether the participants stayed within or crossed the mission and spatial boundaries during VR encoding, we used a linear mixed model (LMM) implemented in R 4.3.1 (R Core Team, 2023)^42^. The packages of lme4 (v1.1-34)^43^ and emmeans (v1.8.7)^44^ were used to fit the models, perform post-hoc tests, and obtain the estimated marginal means for each condition. The lmer package^45^, which extends lme4, was used to provide *p*-values using Wald-type tests for fixed effects and random effects. To examine response accuracy, we used the generalized linear mixed effects model (GLMM) with logistic regression, given the binary nature of the responses (correct or incorrect). To examine confidence in correct responses, we used the linear mixed effects model (LMM).

All models included the fixed factors of mission boundary crossing (Mission boundary) (within vs. across) and spatial boundary crossing (Spatial boundary) (within vs. across) and their interaction, as well as the block order (blocks). Additionally, the models included the random effects of individual participants (subID) with the nested random factors of object identity (objectID). In the recency discrimination test, two consecutive objects from the encoding sequence appear on the same screen. Except for the first object, which was used only as a target, and the last object, used only as a distractor, the other objects were used both as targets and distractors. Thus, each object appeared twice in the memory test: once as a target and once as a distractor. This repetition of the objects could boost memory performance because the response in the first presentation could be memorized, and the object order in the second presentation could be inferred from that memory. To control the possible repetition effect, the occurrence of an object as a target and a distractor in each trial was coded as 1 and 0, respectively. This “repetition” factor was included in the model as a random effect.

## Data Availability

All datasets from this study are available is an open repository at https://osf.io/9k5u6/. It includes the DIY components of the VR project, including 3D models (objects, posters, room), object images. The customer characters used in our study were obtained from the Unreal Engine Store (https://www.fab.com/category/3d-model/characters-creatures).

## Code availability

The codes used in this study, including the PsychoPy program for the memory test and R scripts for data analysis and figure generation, are an open repository at https://osf.io/9k5u6/.
